# Deep Learning-Based Driver’s Hands on/off Prediction System Using In-Vehicle Data

**DOI:** 10.3390/s23031442

**Published:** 2023-01-28

**Authors:** Hyeongoo Pyeon, Hanwul Kim, Rak Chul Kim, Geesung Oh, Sejoon Lim

**Affiliations:** 1 Graduate School of Automotive Engineering, Kookmin University, Seoul 02707, Republic of Korea; 2 Steering Control Logic Engineering Cell, Hyundai MOBIS Technical Center, Yongin-si 16891, Republic of Korea; 3 Department of Automobile and IT Convergence, Kookmin University, Seoul 02707, Republic of Korea

**Keywords:** hands on/off, autonomous vehicle, deep learning, data collection system, state transition

## Abstract

Driver’s hands on/off detection is very important in current autonomous vehicles for safety. Several studies have been conducted to create a precise algorithm. Although many studies have proposed various approaches, they have some limitations, such as robustness and reliability. Therefore, we propose a deep learning model that utilizes in-vehicle data. We also established a data collection system, which collects in-vehicle data that are auto-labeled for efficient and reliable data acquisition. For a robust system, we devised a confidence logic that prevents outliers’ sway. To evaluate our model in more detail, we suggested a new metric to explain the events, considering state transitions. In addition, we conducted an extensive experiment on the new drivers to demonstrate our model’s generalization ability. We verified that the proposed system achieved a better performance than in previous studies, by resolving their drawbacks. Our model detected hands on/off transitions in 0.37 s on average, with an accuracy of 95.7%.

## 1. Introduction

Since the late 19th century, autonomous driving has been deeply studied with the success of deep learning and the improvement of various sensors. The autonomous driving is the most alluring technology to not only the traditional vehicle makers but also to arising Information Technology(IT) companies. The Society of Automotive Engineers (SAE) defined autonomous driving as six levels (zero to five) [1]. Levels zero to two correspond to the driving assistance, in which the driving responsibility lies with the driver; levels three to five correspond to autonomous driving, in which the driving responsibility lies with the system. In December 2021, the Mercedes-Benz group was certified as a level three autonomous driving system from the German Federal Automobile Transport Administration (KBA; Kraftfahrt-Bundesamt) based on the UN-R157 standard, and was the first international standard to define a level three system [2]. Auto Pilot, one of the most famous autonomous driving systems of Tesla, corresponds to level two of the SAE levels. Accordingly, almost all autonomous driving functions all over the world are at level two now, and the level two function is also known as the Advanced Driver Assistance System (ADAS). It provides drivers some convenience or prevents accidents in many situations, by containing the Lane Following Assist (LFA), Highway Driving Assist (HDA), etc. At level two, all ADAS functions are designed to be used as hands on, but some drivers misuse them as hand off. For safety, the United Nations created UN regulation 79, whichforces vehicles to sound an alarm to the drivers when they are “hands off” [3]. The consecutive warning procedure is as follows. The vehicles will notice the driver’s hands off the wheel and provide an optical warning with pictorial information after 15 s at the latest. Then, if the driver does not grip the steering wheel rim after 30 s at the latest, some parts of the optical information must turn red and the acoustic warning signal should be provided. Lastly, at the last 30 s after the acoustic warning has started, the ADAS function must be automatically deactivated. After deactivation, the vehicle must inform the driver about it by a new acoustic signal for at least 5 s. Meanwhile, some ADAS functions operate differently when the driver drives and when the system drives. Therefore, the vehicle should notice whether the driver wants to drive on his/her own. The driver might feel uncomfortable if the system intervenes when he/she is driving and it could be dangerous due to the unwanted maneuver. In addition, following the SAE levels, the driver is not obliged to monitor the driving situation at level three. However, the driver has to take control immediately if an emergency occurs. At the same time, the autonomous vehicle has to give control to the driver as soon as it notices the driver’s intervene intention. Therefore, the accurate hands on/off detection (HOD) is required for the ADAS control at the current autonomous driving level.

Early studies mainly utilized the driver’s steering torque applied to the steering wheel when the driver grips it. Moreillon et al. [4] estimated the torque using the torsion bar sensor of the electric power steering (EPS) and angle encoder of a motor to consider the torque solely generated by the driver, except for the torque generated by the road surface. Schinkel et al. [5] estimated a transfer function between the EPS torque and steering column and decided the driver intervention on the simulator. Differently from other studies, which only considering the driver torque, Wang et al. [6] decided the driver intervention in a Lane Keeping Assist (LKA) situation, in which the driver torque and LKA function’s torque were combined. Moreillon et al. [7] and Xing et al. [8] predicted the driver’s driving intention and suggested various goals under several driving scenarios.

Some studies directly measured the driver’s torque by attaching sensors to the steering wheel rim. Li et al. [9] used a strip-shaped flexible grip sensor to measure driver fatigue. Muhlbacher-Karrer et al. [10] used inkjet-printed electrodes on a bendable substrate to detect where the hands touch and capacitance. They modeled a optimal electrode structure based on the finite element simulation method. Chen et al. [11] utilized nine-axis sensor readings from a wearable sensor and its bluetooth-paired smartphone. They divided the steering wheel into a 12 o’clock position and figured out where a hand is placed based on the static hand location information and dynamic rotation information. However, these sensor-based systems might not work properly if the driver is wearing gloves.

With the development of camera sensors, computer vision and deep learning, camera image-based studies have been conducted. Johansson et al. [12] detected the driver’s hand and steering wheel from camera images using a convolutional neural network (CNN) and long short term memory (LSTM). The camera is mounted above the driver head; thus, this system is vulnerable underneath the steering wheel. Hoang et al. [13] and Rangesh et al. [14] also used camera images to detect whether the driver is holding a steering wheel. They constructed their network based on the traditional CNN, such as Faster R-CNN [15]. Yudkin et al. [16] utilized synthetic photo-realistic in-cabin data when few real data are available. Expanding the research area, some studies are conducted to detected driver distraction, including hands on/off detection from images [17,18,19]. Meanwhile, to overcome the weakness of camera images, such as the low reliability to illuminance changes, Borghi et al. [20] utilized infrared images instead of RGB ones.

Torque estimation logic is relatively simple and easy to compute but does not guarantee precise torque and needs to set a certain threshold. It might also need to newly set depending on the drivers or vehicle types. Utilizing the attached sensors can help in detecting more accurately, but it costs. It leads to the increment of expense, and attempts to use other sensors on the steering wheel, such as bio sensors. In addition, there is a fatal disadvantage, where the HOD function cannot be used at all until a new sensor is replaced if a sensor breaks. Camera image-based approaches have advantages of using images and deep learning. Images have more information than the torque and sensors on the steering wheel, and deep learning can help more precise modeling. However, it has the same disadvantages of sensors on the steering wheel, such as high cost, breakdown and inherent disadvantages of the camera itself. Moreover, labelling the image manually has a high cost, and very precise labelling is needed for training a model. One more important aspect is that the above-mentioned studies evaluated their system on typical metrics such as accuracy, precision and recall. Considering the actual driving, drivers are holding or not holding the steering wheel almost all the time, with only a short moment of state transitions. However, the model’s wrong detections mainly occur when the hands on/off state changes. Therefore, traditional metrics have limits to explain hands on/off detection events. A new metric, which describes how accurately and how fast a model detects the state transition, is needed. Therefore, to overcome all those limitations, we propose a deep learning-based driver’s hands on/off detection model using in-vehicle data from the controller area network (CAN). This system is cost-free and less likely to break down, as it does not need any extra sensors. It is also robust, because in-vehicle data are measurable independent of external conditions. Moreover, we established a data collection system, which stores time-synced in-vehicle data and whether a driver is holding the steering wheel. Additionally, we defined a new metric considering the hands on/off state change for a comprehensive understanding of driving to evaluate our model. The main contributions of this study are:

- A data collection system that we made can help save significant time and cost to prepare data for training a deep learning model, and supervised by making data to be collected with labels. It guarantees high precision and can be utilized to similar tasks with a few modifications.
- We utilized in-vehicle data instead of any extra sensors. It helps us to reduce development costs and to make a system more reliable.
- The proposed new metric, considering the state transition, helps to understand the model’s performance in a more comprehensive way.
- Our model is validated in that it also works well for the new drivers. It can be used universally by the new drivers with few drivers’ data.

The rest of this paper is structured as follows. Section 2 gives a description of a data collection system that we built, dataset and preprocessing methods. Section 3 provides the description of the model structure of our deep neural network, the confidence logic and the evaluation metrics. In Section 4, the experimental setup and results are presented. Finally, the conclusion of our work and directions for future work are presented in Section 5.

## 2. Dataset

In image-based studies, the researchers of [12,13,14,16,17,18] spent significant time labelling their image data. This requires the high cost of human resources and money, and it is challenging to precisely label a confusing image, such as right before touching the steering wheel. It makes it difficult to make use of big data.

### 2.1. Data Collection System

**Figure 1:**
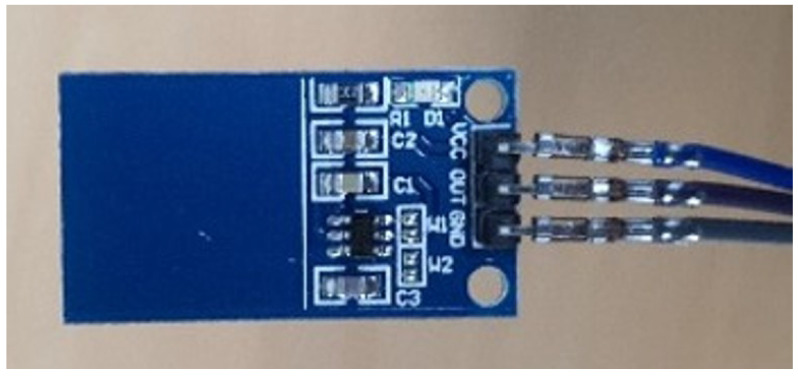
A capacitive sensor.

**Figure 2:**
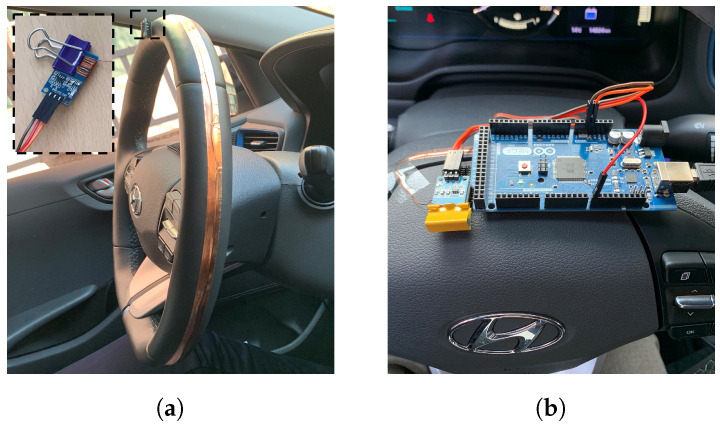
Data collection system. (**a**) The steering wheel covered with copper wire and tape; (**b**) a capacitive sensor connected to an Arduino board.

We established a data collection system using a capacitive sensor in Figure 1 to store the time-synced in-vehicle data and ground truth. A capacitive sensor measures a tiny current from a driver’s hand so it can be used as a ground truth for whether a driver’s hand is on the steering wheel in the in-vehicle data. However, we could not cover the whole steering wheel with that because it is square-shaped and very small. Therefore, we added a copper wire and tape to enlarge the contact area. A copper wire and tape covered a steering wheel, and a copper wire was connected to a capacitive sensor at one end, as shown in Figure 2a. A wire mainly helps in conveying a current to the capacitive sensor, and a tape enlarges the contact area. A capacitive sensor is connected to an Arduino board (Figure 2b) and measures a current as positive (1) or negative (0) for each hands on/off. The in-vehicle data was measured by CANape application and a VN1630A device of Vector [21]. Both in-vehicle data and the hands on/off label is recorded in a python program via serial communication and the win32com library [22]. These data are stored every 10ms in a time-sync (under 1 ms difference) by comparing their timestamp. This data collection system is needed only when we collect data for training a model, and every vehicle on a road can make use of our HOD model without this system. This system is very economical because a sensor and a board is cheap, and is powerful because we do not need a labelling process at all. It can also be used or extended easily for other studies using in-vehicle data and hands on/off data.

### 2.2. Data Description

**Figure 3:**
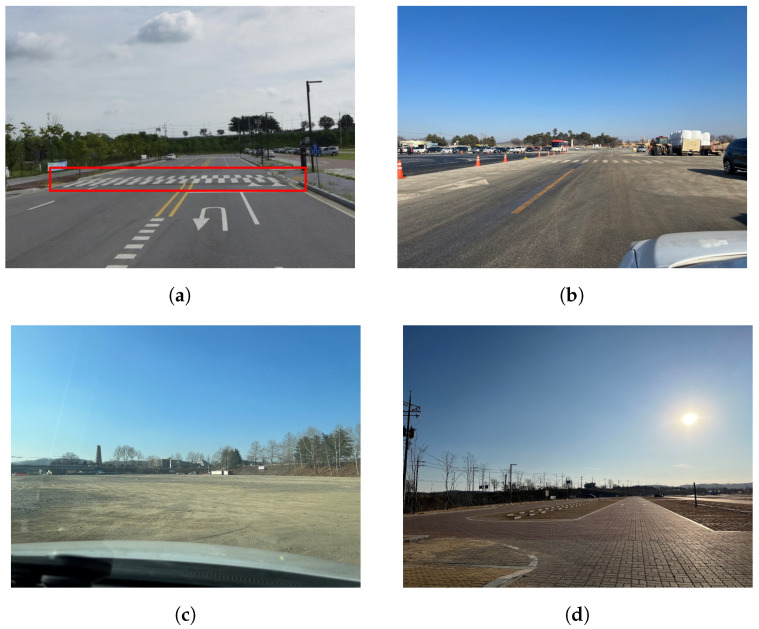
Data collection roads. (**a**) An asphalt road with a bump (red rectangle); (**b**) an asphalt road; (**c**) an unpaved road; (**d**) a sidewalk blocked road.

Figure 3 shows where we collected data. We searched for some low-traffic roads because driving without holding the steering is dangerous. To discern for in-vehicle data variance between the hands on the steering wheel and from a rough surface, asphalt roads, bumps, sidewalk-blocked roads and unpaved roads are included. The deep learning model does nit need to discern between these types of roads, but has to capture the variances of the hands on/off states according to the road types. Data were mainly collected under a soft steering scenario with a real vehicle. The speed varies from 30 kph to 90 kph. A single drive is about a minute and is comprised of 4∼6 consecutive hands on/off changes, on average. A total of 3 drivers from ages 26 to 30, who have driving experiences of more than 5 years, participated in the study. About 5 h and 25 min of valid data were collected, except in low-speed situations (under 5 kph), as shown in Table 1. Data collected under 5 kph were discarded, since we defined the “driving” state as above 5 kph, considering vehicle dynamics. Since we drove only on the safe roads, the length of a single drive was not long enough. Therefore, we had to spend much more time driving than the actual total data length shows (about 5.5 h). However, balancing the data for classification was made relatively easy by repeating a short road several times.

**Figure 4:**
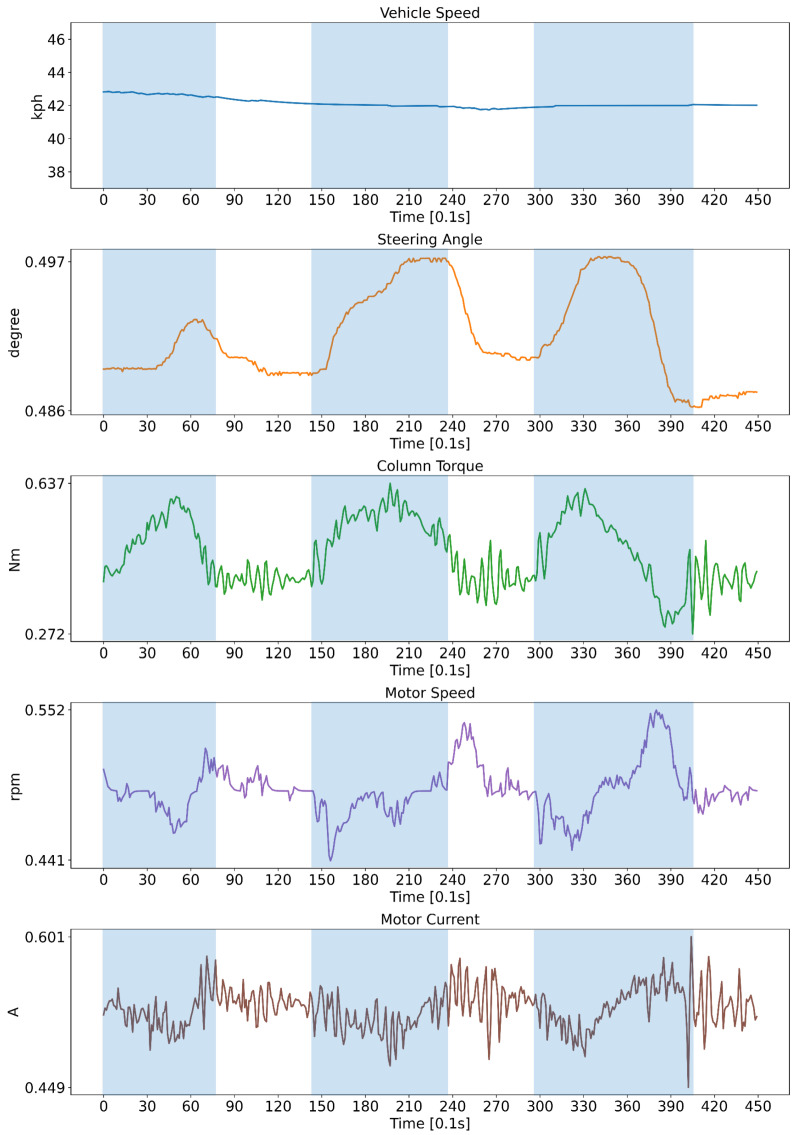
An example of collected data. Hands on and hands off state was shown as blue and with transparent background, respectively. All signals except for vehicle speed were min-max normalized.

The input signals of a model is as shown in Table 2. We selected four signals highly related to the steering, in order to detect the hands on/off. We also wanted a lightweight model to embed an electronic control unit (ECU); thus, we selected only a few signals. The output of our model is a probability of the hands on (Table 3). Since we defined 1 as hands on and 0 as hands off, the output value can be interpreted as a “hands on” probability. Figure 4 shows an example of input signals. The blue and transparent background means the hands on state and hands off state, respectively. These data were collected while driving around at 43 kph with 5 state transitions.

### 2.3. Data Preprocessing

Data were split into a train, validation and test set of a ratio of 0.6, 0.2, and 0.2, respectively, following common practice. Each dataset keeps the same proportion of roads and drivers as the total data. A few missing values can be observed as the data frequency being too fast. Since there are no drastic changes within 10ms, the missing values were replaced with the previous value. Then, data were resampled to 10 Hz, which is enough to detect signal changes, and each signal was normalized between 0 and 1 to have the same importance between signals. Lastly, the time window of the 100ms stride is applied for the LSTM input.

## 3. Proposed System

In this chapter, the proposed system is introduced. The architecture of a model and a confidence logic for the robust output are discussed. When it comes to the evaluation metrics, we propose a new HOD metric, considering the state transition.

### 3.1. Model Architecture

**Figure 5:**
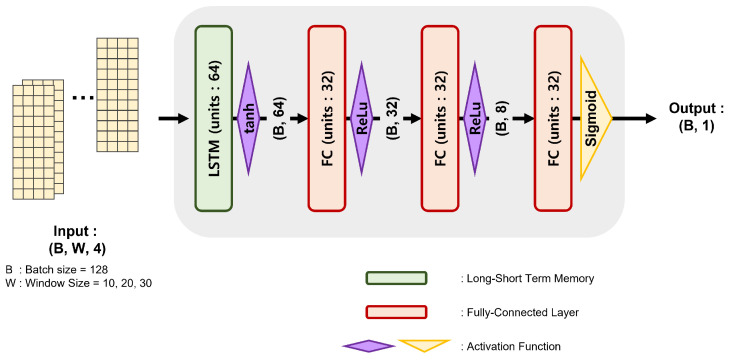
A diagram of proposed network and data.

We had two options to implement the hands on/off detection function, machine learning and deep learning. Although machine learning also demonstrates a good performance on the binary classification problem, deep learning is advantageous because of its scalability and generalization. Therefore, we chose the deep learning-based approach. We used a long short term memory (LSTM) [23] layer to handle the time series data. We also intended a lightweight network to be advantageous for embedding in the in-vehicle system with the limited computational resource. Therefore, only one LSTM layer with 64 neurons is used as an input layer, then, fully connected (FC) layers are followed. The FC layers gradually lessen the neurons with relu activation, and the output FC layer has one neuron with the sigmoid activation. The sigmoid activation gives an output between 0 and 1, and it could be interpreted as a probability. The full architecture is summarized in Table 4 and depicted as a diagram in Figure 5. B and W mean batch size and window size, respectively. We used 128 as the batch size and 10, 20 and 30 as the window size in the experiments.

The cross entropy was used as a loss function. In Equation (1), *y* denotes the ground truth (0 or 1) and *p* denotes the model’s output probability. The cross entropy function measures the difference between the true probability distribution and model’s prediction distribution and is minimized during training. The Adam optimizer [24] is adopted to minimize the loss because it performs well in common classification problems.

(1) crossentropy=−(ylog(p)+(1−y)log(1−p))

### 3.2. Confidence Logic

**Figure 6:**
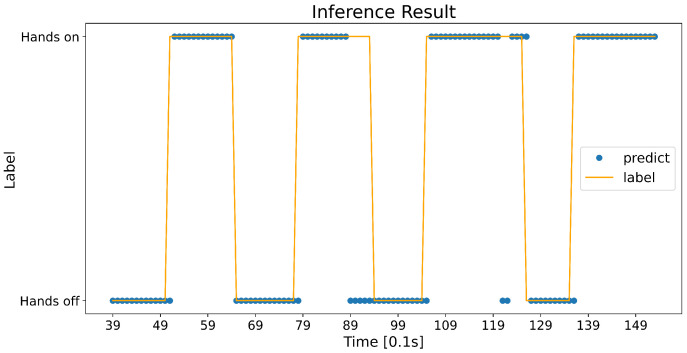
Raw predictions of a network.

The confidence logic is a kind of postprocess applied to the predictions of a network. The raw predictions of a network are somewhat unstable. Figure 6 shows an example of the raw prediction by a threshold of 0.5, that is, the probability over 0.5 is hands on and the other is hands off. Some outliers can be observed around 12 s. It might be a slight loss when it comes to the entire accuracy, but it can be a crucial error in terms of the state transition and its maintenance. Therefore, we devised a confidence logic for a robust prediction, as follows. To avoid a quick state transition by outliers, the recent 3 model’s ouput probabilities (for 0.3 s) are used for a prediction (Equation (2)). Then, the hands on/off state is determined by comparing a mean of those probabilities to the upper and lower thresholds (Equation (3)). The upper and lower thresholds are set after the testing of different values and designed to more quickly detect a transition from hands on to hands off.

(2) $$O_{t}=\frac{1}{3}\sum_{n=0}^{2}o_{t-n}$$

(3) $$\begin{array}{c} \left(\begin{array}{cc} hands\;on & if\;\;0.6<=O_{t}\\ previous\;state & else\;if\;\;0.45<=O_{t}<0.6\\ hands\;off & else \end{array}\right. \end{array}$$

**Figure 7:**
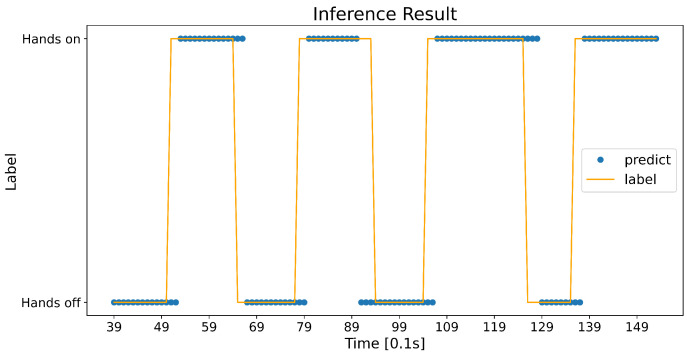
Predictions of a network with a confidence logic.

Figure 7 shows the confidence logic applied to the result in Figure 6. Outliers around 12 s were adjusted to remain on the correct label. A short delay also occurred when the state changed because an effect of the current probability was lessened. However, a slower and but stable detection is more desirable than a quick but fluctuating detection.

### 3.3. Evaluation Metric

**Figure 8:**
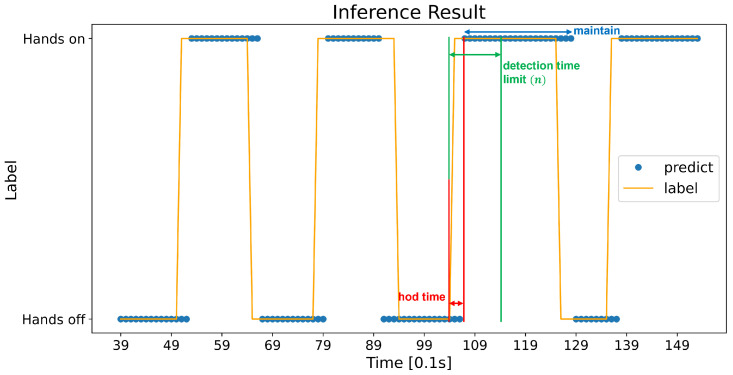
An example of calculating the HOD metric. The ground truth changes at 10.4 s and our model detected it at 10.7 s. The time difference between them is HOD time. If it is shorter than the detection time limit and maintains for at least a second, it is regarded as a correct detection.

We evaluated our model on two metrics: (1) Common classification evaluation metric; (2) HOD metric. The former means the accuracy, precision, recall, f1 score and area under the curve (AUC), which are all commonly used to evaluate a classifier. Considering the HOD problem, however, the model’s output being fast and stable is more important. These metrics cannot explain this aspect. Therefore, we newly defined that the “HOD metric” comprises the HOD accuracy and HOD time (Figure 8). A HOD accuracy is calculated based on when the state changes, and not every data sample is 0.1 s. We calculated this HOD accuracy according to the “detection time limit”, which is different from the inference time. Regardless of how fast the model performs its calculation (inference), when the model is detecting, the transition can vary. If the model detected a transition after 10 s from when it really occurred, it should not be considered as a right answer. Therefore, we intended a fair evaluation by setting the detection time limit. If the model detects the transition within *n* seconds (detection time limit) and maintains it more than a second, it is regarded as a right answer. At this moment, the difference between when the actual transition occurs and when the model detects it is defined as “HOD time”. The HOD time is suggested with a mean and standard deviation. Both the HOD accuracy and HOD time is as demonstrated in Equations (4)–(8). $t_{hod}^{i}$ means the *i*th time difference between when the actual transition occurs ($t_{gt}^{i}$) and when the model detects ($t_{detect}^{i}$) (Equation (5)), and $T_{HOD}$ (Equation (6)) is a set of those time differences.

(4) $$HOD\;accuracy\;=\;\frac{Number\;of\;correct\;detections\;on\;state\;transition}{Total\;number\;of\;state\;transitions}$$

(5) $$\begin{array}{cc} t_{hod}^{i}\; & =\;|t_{gt}^{i}\;-\;t_{detect}^{i}| \end{array}$$

(6) $$\begin{array}{cc} T_{HOD}\; & =\;\{t_{hod}^{1},\;t_{hod}^{2},\;\ldots ,\;t_{hod}^{n}\} \end{array}$$

(7) $$\begin{array}{cc} HOD\;time\;mean\; & =\;\bar{T}\;=\;\frac{1}{n}\sum T_{HOD} \end{array}$$

(8) $$\begin{array}{cc} HOD\;time\;standard\;deviation\; & =\;\sqrt{\frac{\sum_{i=1}^{n}{\left(t_{hod}^{i}-\bar{T}\right)}^{2}}{n}} \end{array}$$

## 4. Experiments and Results

In this section, we elaborately discuss the experiments we designed, in order to evaluate the performance and robustness of the proposed model.

### 4.1. Implementation Details

Our proposed system was implemented with the Keras 2.7 version, TensorFlow [25] 2.7 version and Python 3.8 version. The training was carried out using the NVIDIA GeForce RTX 3080 Ti GPU with an Intel i9-12900K CPU and 128GB of RAM on the Ubuntu 20.04 OS. To avoid overfitting, the early stop was adopted, in which the validation loss was monitored. The training was stopped when the validation loss was not reduced any more than 20 epochs. The Adam optimizer was used to train the model with a batch size of 128, with 0.001 of the learning rate.

### 4.2. Training Results

The training was conducted on a time window size of 10, 20 and 30. This means that the input data time length is 1, 2, 3 s, respectively, because the data were sampled every 100 ms. Table 5 demonstrates the results on the test set according to each window size. Our model demonstrates great performance on all time window sizes. The overall best result was found in 10, although the recall was the highest in size 20. In general, touching or releasing the steering wheel occurs within 1 s; thus, it can be interpreted that 1 s input is enough. We also can speculate that our model guarantees a stable performance on all situations, as both precision and recall are high with little gap. All of the following results were obtained from an optimal model trained with the time window in size 10.

**Figure 9:**
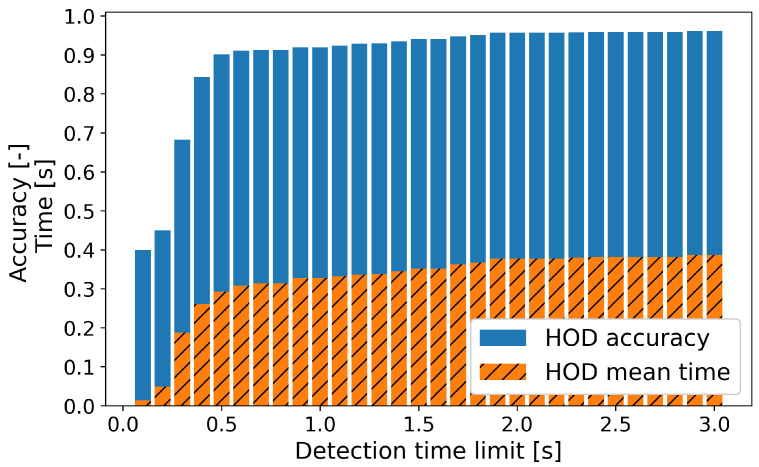
HOD accuracy and the mean HOD time according to the detection time limit on the test set.

Table 6 shows the HOD performance of our model. The accuracy of detection, mean and standard deviation of the detection time are calculated considering every hands on/off transition with various time limits. The accuracy within the time limit of 1 s is 92.34%, which is higher than the simple accuracy of 86.57% from Table 5. It describes that the commonly used metrics on the classification cannot explain the HOD events enough. Our model detected the hands on/off transition quickly with a small deviation, and both HOD accuracy and HOD time are increased as expected when the detection time limit is increased. Table 7 shows the results of Table 6 divided into situations of hands off → on and hands on → off, respectively. Our model detected the hands on → off change better in terms of accuracy. The mean time required for the detection was longer in the case of the hands on → off, which is an acceptable result, considering that the driver generally exerts more force when holding the steering wheel. Since it takes longer to determine the situation of the hands on → off, the threshold of the hands off in confidence logic (Equation (3)) was set to 0.45, and is slightly higher than 0.4 for a faster detection. However, the standard deviation of the HOD time is small in both cases; thus, it can be assumed that both cases have a stable distribution for the detection time. Figure 9 describes the HOD accuracy and the mean HOD time according to the detection time limit with a 0.1 s interval. After 0.3∼0.4 s, which is around the mean time, the HOD accuracy was increased drastically. Moreover, the few increases on the accuracy and mean time after 2 s can be observed.

**Figure 10:**
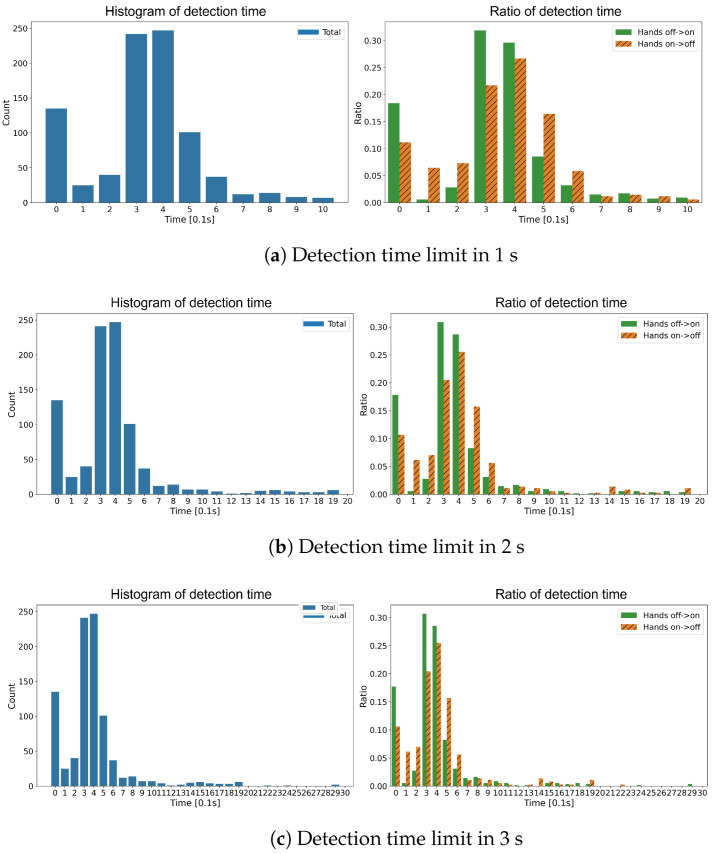
Detection time histogram according to the detection time limit. Ratio of detection time was represented on the right figure.

Figure 10 describes the detection time according to the time limit and state transition. The left figure is a histogram of the total state transition and the right figure shows the ratio of each case from the left. The most transitions were detected in 0.3∼0.4 s, which is close to the mean, and the further from the mean, the less samples were there. In addition, many transitions were detected as soon as they actually changed. We can assume that some transitions were easy to detect because the rapid holding or releasing of the steering wheel creates a rapid change in signals. Moreover, we can notice that the ratio of the hands off → on is higher before the mean time, and is lower after the mean time. Grasping the steering wheel usually introduces more change in signals, and it leads to the relatively faster detection. Lastly, almost all detections were made within 2 s (Figure 10c). This is fast enough to sound alarms at the right time to the drivers in compliance with the UN regulation, as discussed in Section 1.

Table 8 compares the accuracy and AUC of previous studies to our method. Early studies, which utilized the driver’s torque, did not suggest quantitative performances. As far as we know, moreover, there was no research that evaluated their method similarly to us (HOD metric). Although our model demonstrates a lower “accuracy”, it demonstrates the highest performance when it comes to the “HOD accuracy”. Furthermore, our studies can explain the HOD event more comprehensively.

### 4.3. Driver Generalization Results

One more important aspect that a deep learning model must have is a generalization ability. If a model fails to be generalized, it can be only used under the trained conditions. Therefore, we evaluated our model’s generalization performance with the new drivers. For this evaluation, we additionally acquired the data of an hour from two expert drivers who were not involved in the data collection before training. In addition, by collecting data including some new roads, we designed the generalization performance for new roads, which is also examined.

Table 9 shows the results of the two new drivers. For driver A, it demonstrated a high performance in the window size of 30 as well as in the size of 10. Overall, however, a window size in 10 demonstrated the best performance. Some metrics, such as accuracy, demonstrated even better performance than on the test set (Table 5). A clear and important aspect is that our model worked well for the new drivers. Table 10 and Table 11 describes the HOD performance of our model for the new drivers. When the detection time limit is one second, the HOD accuracy was about 92.3% on the test set. The HOD accuracy on both new drivers was very close to that. Moreover, when the detection time limit is longer than one second, the accuracy for the new drivers was almost the same as the results on the test set. However, a little more time was needed for the accurate detection for the new drivers. Meanwhile, hands off → on changes were detected 0.1∼0.2 s faster than hands on → off changes. This difference between the two state changes is slightly longer compared to the difference between the two state changes of less than 0.05 s on the test set. We surmise that this comes from the difference between the driving habits of the drivers. Another possible cause comes from having less variety of data, because we collected new driver data in one day. Collecting more data for a longer period and from more drivers is planned to be conducted in a future work.

**Figure 11:**
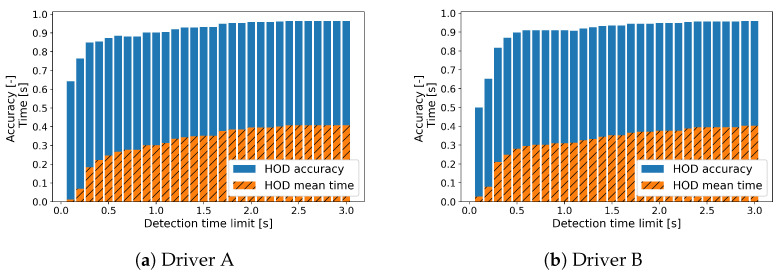
HOD accuracy and the HOD mean time according to the detection time limit on new drivers.

Figure 11 describes the HOD accuracy and the mean HOD time according to the detection time limit with 0.1 s interval for the new drivers. Similar to the test set (Figure 9), there is an drastic increase in the HOD accuracy, of around 0.3 s, and almost the highest accuracy at 2 s.

## 5. Discussion

We verified that driver’s hands on/off detection is possible with a deep learning model using in-vehicle data. Our model detected hands on/off transitions in 0.37 s, on average. Since in-vehicle data are time series data, our model, which contains an LSTM layer, worked well on this problem. A precise data collection system using a capacitive sensor and an Arduino board helped the model learn unique characteristics of the hands on and hands off state. Moreover, a confidence logic helped the model make its output more robust. Our system can be utilized easily in other studies using in-vehicle data and is especially effective for supervised learning.

## 6. Conclusions and Future Work

Hands on/off detection is a crucial task for current autonomous vehicles. Most previous works had at least one distinct drawback of their own. In this study, we proposed a deep learning network that utilizes steering data. This approach is almost zero cost and reliable, since steering data can be measured easily from built-in sensors. For training, we established a data collection system, which enables auto-labelling when collecting steering data. A new evaluation metric was introduced and experiments were carried out to demonstrate the performance and robustness of our model. In addition, we evaluated our model on the new drivers to ensure the generalization power. We verified that our architecture can effectively resolve the drawbacks of the previous studies and can achieve better performance. Considering real driving with other vehicles, the hands on/off can depend on surrounding events. For this, we also purposely collected new drivers’ data on new roads, apart from the roads initally used in the experiment (Figure 3). Our model can handle various real driving situations, as we have observed in Table 9, Table 10 and Table 11.

Although we demonstrated the generalization ability with the new drivers, we could not fully explain why the detection time differed between the hands off → on and hands on → off state transitions. This is going to be discussed in our subsequent research. Our future work also may include various vehicle types, not only the hybrid sedan we used but also sports utility vehicles, trucks and electric vehicles. Lastly, we intended a lightweight network for utilizing it on the ECUs of a vehicle. Model compression and embedding after selecting a target ECU will be conducted.

## Figures and Tables

**Table 1:** Summary of collected data. Total data are about 5 h and 35 min, and were well balanced.

Road Type	Label	Data Size (Proportion)
Asphalt	Hands on	1 h 20 m (55.91%)
	Hands off	1h 3m (44.09%)
Unpaved/Blocked	Hands on	1 h 48 m (58.96%)
	Hands off	1 h 15 m (41.04%)

**Table 2:** Input data of a deep learning model.

Input Data	Minimum Value	Maximum Value	Unit
Steering wheel angle	−466.0	486.4	deg
Steering wheel column torque	−4.6	6.0	Nm
Steering motor speed	−1413.8	1459.4	rpm
Steering motor current command	−53.1	46.3	A

**Table 3:** Output data of a deep learning model.

Output Data	Minimum Value	Maximum Value	Unit
Hands on probability	0	1	-

**Table 4:** HOD model structure.

Layer	Neuron	Activation Function
LSTM	64	Tanh
FC	32	Relu
FC	8	Relu
FC	1	Sigmoid

**Table 5:** Evaluation results on the test set. End epoch of training means the last epoch at training stopped by the early stop regularization.

Time Window Size	End Epoch of Training	Accuracy	Precision	Recall	F1 Score	AUC
10	97	0.8657	0.9027	0.8702	0.8862	0.9355
20	70	0.8631	0.8862	0.8826	0.8844	0.9319
30	65	0.8564	0.9008	0.8487	0.874	0.9293

**Table 6:** HOD accuracy and time on the test set (time window size = 10). The unit detection time limit and HOD time is second.

Detection Time	HOD Accuracy	HOD Time	
Limit (*n*)		Mean	Standard Deviation
1	0.9234	0.3323	0.1985
2	0.9574	0.3774	0.3058
3	0.9617	0.3873	0.3395

**Table 7:** HOD accuracy and time considering state transition on the test set (time window size = 10). The unit of detection time limit and HOD time is second.

State Transition	Detection Time	HOD Accuracy	HOD Time	
	Limit (*n*)		Mean	Standard Deviation
Hands Off → On	1	0.9197	0.323	0.1998
	2	0.9494	0.3621	0.2963
	3	0.9546	0.3751	0.3439
Hands Off → On	1	0.9292	0.3466	0.1958
	2	0.97	0.4008	0.3184
	3	0.9728	0.4059	0.3319

**Table 8:** Comparing the performance of some methods. Ref. [16] suggested AUC for left/right hand, respectively. All figures were cited from their papers.

Type	Method	Accuracy	AUC	HOD Accuracy
Wearable sensor	[11]	91.59%	-	-
Image data	[13]	93%	-	-
	[16]	-	0.9369 / 0.9530	-
In-vehicle data	ours	86.57%	0.9355	95.74%

**Table 9:** Evaluation results on the new drivers’ data.

Driver	Time Window Size	Accuracy	Precision	Recall	F1 Score	AUC
A	10	0.8851	0.8983	0.9193	0.9087	0.9358
	20	0.8748	0.8931	0.9036	0.8984	0.9324
	30	0.8857	0.9048	0.9123	0.9085	0.9402
B	10	0.9009	0.9018	0.9271	0.9143	0.9488
	20	0.8979	0.9131	0.9051	0.9091	0.9507
	30	0.8857	0.9048	0.9123	0.9085	0.9402

**Table 10:** HOD accuracy and time on the new driver data (time window size = 10). The unit of detection time limit and HOD time is second.

Driver	Detection Time	HOD Accuracy	HOD Time	
	Limit (*n*)		Mean	Standard Deviation
A	1	0.9053	0.3131	0.2194
	2	0.9586	0.3954	0.3692
	3	0.9645	0.4074	0.3985
B	1	0.9068	0.312	0.1787
	2	0.9472	0.3757	0.3111
	3	0.9596	0.4023	0.3876

**Table 11:** HOD accuracy and time considering state transition on the new driver data (time window size = 10). The unit of detection time limit and HOD time is second.

Driver	State Transition	Detection Time	HOD Accuracy	HOD Time	
		Limit (*n*)		Mean	Standard Deviation
A	Hands Off → On	1	0.8913	0.2268	0.1798
		2	0.9674	0.3242	0.3852
		3	0.9728	0.3358	0.4141
	Hands Off → On	1	0.9221	0.4127	0.2188
		2	0.9481	0.4822	0.3285
		3	0.9545	0.4946	0.3599
B	Hands Off → On	1	0.9153	0.2704	0.1579
		2	0.9322	0.2994	0.2453
		3	0.9379	0.3108	0.2854
	Hands Off → On	1	0.8966	0.3638	0.1893
		2	0.9655	0.4657	0.3535
		3	0.9862	0.5084	0.4573

## Data Availability

Not Applicable.
